# Motivating medical students to do research: a mixed methods study using Self-Determination Theory

**DOI:** 10.1186/s12909-015-0379-1

**Published:** 2015-06-02

**Authors:** Sara K. Rosenkranz, Shaoyu Wang, Wendy Hu

**Affiliations:** 1 School of Medicine, Medical Education Unit, University of Western Sydney, Campbelltown, NSW Australia; 2 Department of Human Nutrition, Kansas State University, 212 Justin Hall, Manhattan, KS 66506 Kansas

**Keywords:** Undergraduate medical education, Student research, Motivation, Competency-based training, Curriculum design, Evidence based practice, Self-determination theory, Mixed methods research

## Abstract

**Background:**

It is widely accepted that all medical graduates should understand the uses and methods of rigorous research, with a need to promote research to graduates who will pursue an academic career. This study aimed to explore, identify and explain what motivates and demotivates medical students to do research.

**Methods:**

A convergent parallel mixed methods study was conducted. Cross-sectional quantitative survey data (n = 579) and qualitative semi-structured interview findings (n = 23) data were separately collected and analysed. Informed by Self-Determination Theory (SDT), quantitative and qualitative findings were integrated to develop a model for the factors associated with medical students’ expressed motivation to do research, and related to clinical and research learning activities at different stages in an undergraduate medical program.

**Results:**

Only 7.5 % of students had research experience prior to entering the program. Survey results revealed that students who had experienced exposure to the uncertainties of clinical practice through clerkships (Pre-Clinical (48 %) vs Clinical Years (64 %), *p* < 0.001), and a sense of achievement through supported compulsory research activities which were conducted as a team (Pre- Community Research (51 %) vs Post-Community Research (66 %), *p* < 0.001), were more likely to view future research activities positively. When integrated with qualitative findings using the three SDT domains of *autonomy*, *competence* and *relatedness*, eight major themes were identified: Self & Time, Career, Bureaucracy, Financial, Confidence, Clinical Relevance, Research as a Social Activity, and Personal Relevance. The findings suggest that motivation to do research is associated with increasing internalization of intrinsic motivators; in particular those associated with *competence* (Confidence) and *relatedness* (Clinical Relevance, Research as a Social Activity).

**Conclusions:**

SDT is useful for understanding the motivation of individuals and how curriculum can be designed to optimise motivation. Study findings suggest that well supported compulsory research activities that incorporate group learning and elements of choice may promote motivation to do research, and potentially, careers in research, even in a research naive student body.

**Electronic supplementary material:**

The online version of this article (doi:10.1186/s12909-015-0379-1) contains supplementary material, which is available to authorized users.

## Background

An understanding of the uses and methods of rigorous research is necessary for all medical graduates, as future users of research for clinical decisions, translators of research to clinical practice, and as communicators of research to patients and colleagues [1–3]. There is also a need to attract and support students who will become clinician scientists or academic physicians [4], with fewer choosing research careers, and clinician researchers being referred to as an “endangered species” [5–10].

Curriculum strategies to promote research pathways have included: identification and recruitment during medical school [10], early exposure to research experiences [6], teaching on research methodology [11], vacation and elective research activities [12, 13]. These are associated with more positive attitudes towards research, greater acquisition of research skills, and longer term, more publications [9] and uptake of academic careers [14, 15]. While valuable for a subset of students, these strategies may unintentionally exclude students who do not take up additional activities, but have the capacity to become good researchers. Most studies have been conducted with graduate cohorts [14–16], so findings may not apply to more “research naive” students, or students in direct high school entry programs. Mandatory research activities have been recommended [17], but there is little evidence for how these should be designed, to help curriculum leads decide how to trade off other activities in already crowded curricula. Ultimately, decisions about curricular balance depend on the particular mission of a medical school, but little is known about the factors and experiences that could motivate and inspire students to include research in their future careers [18].

To date, findings on student research have tended to be descriptive, and not underpinned by theory. A theoretically informed approach can strengthen the transferability of any findings, and point the way to more effective strategies to motivate students to do research both during and after medical school. Self-Determination Theory (SDT) [19, 20] is a theory of motivation which has been used in: sport and exercise, health and well-being, psychotherapy and education, and more recently, medical education [21]. In SDT, *Autonomy*, or behaviors initiated with a sense of volition and choice [19]; *Competence*, or being effective, optimally challenged, exercising personal capacity and extending skills [19]; and *Relatedness*, or having secure attachments, being involved in caring relationships, and a sense of connection with a group purpose and ideals [19, 21], are psychological needs that when fulfilled, promote positive functioning in the educational context [20, 22]. Previous research has found that environments that promote autonomy (such as by increasing student choice) rather than reducing it increase intrinsic motivation [22]. Further, *external regulation*, a form of controlled motivation, describes acting only to receive an external reward, or avoid a punishment, or comply with social pressure. In contrast, *identified regulation*, a form of autonomous motivation, occurs when an individual identifies highly with the importance or value of a behavior or practice [20, 22]. In the SDT model of change, autonomy serves to facilitate an increased sense of competence, but competence alone is not sufficient to ensure change or adherence. Rather, competence must be associated with increased autonomy. Finally, based on the idea that humans possess the universal need to interact, social environments can positively influence relatedness (such as a sense of belonging) or can be a negative influence, disrupting the processes of growth and integration.

Self-determination is also specific to particular behaviors. Applied to student learning, SDT begins with the premise that students are intrinsically motivated to learn and be challenged intellectually. To remain intact and grow, this innate motivation should be supported by interactions in the social environment that satisfy both intrinsic and extrinsic motivators in the three domains. Over time, the effect of these experiences is internalized so that research, for example, becomes inherently interesting or enjoyable, and students become more intrinsically motivated to continue research, and more likely to take up research as graduates. Conversely, according to SDT, when students perceive that the primary focus of learning is to obtain external rewards, such as exam grades, they can perform more poorly due to a negative effect on intrinsic motivation [23].

Accordingly, this study used a theoretically informed approach to investigate what motivates medical students to conduct research. To provide greater insights than that afforded by surveys or audits alone, a mixed methods study was designed to collect, analyze and integrate quantitative and qualitative data, using SDT as an analytic framework. By better understanding student motivations, a secondary aim was to design more effective and evidence- based strategies to strengthen student interest in research during medical school.

## Methods

**Fig. 1:**
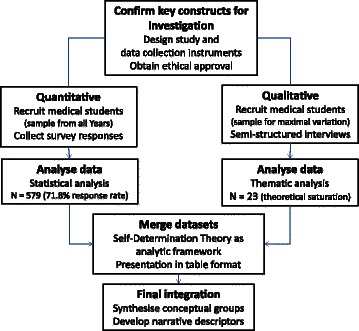
Study Design – convergent parallel mixed methods

A convergent parallel mixed methods study was designed to describe medical student experiences, views and motivations on doing research, and to explore the reasons for these views and motivations [24, 25]. In contrast to a sequential mixed methods approach where qualitative interviews might be aimed at explaining quantitative survey results, and thus interview questions being influenced by survey results, a convergent design collects and analyses quantitative and qualitative data separately, with integration of findings occurring afterwards. This approach gives equal weighting to both qualitative and quantitative data and enhances the potential of the independent datasets to triangulate findings, provided both focus on the same conceptual constructs (see Fig. 1). The study was approved by the Human Research and Ethics Committee of the University of Western Sydney (ID no. H8792).

### Context

The study setting is a recently established medical school (2007) in a public University with an emerging medical research profile. The school offers a direct high-school entry medical program, with fewer than 10 % of students having completed a previous degree. The 5-year program comprises a Pre-Clinical stage (Years 1–2) and a Clinical stage (Years 3–5), when students enter full time clinical clerkships. In Year 4, all students conduct, in groups, a 160-h Community Research (CR) project on a health issue affecting the local community, supervised by a content expert, with research methods instruction from a research academic. Groups develop a research question and project proposal, collect and analyze data and write a report formatted as a journal manuscript. Optional student research opportunities include: vacation scholarships, embedded Honours, an intercalated research year, and doctoral research. There were no significant changes in the research curriculum during data collection.

### Quantitative survey methods

Students in all Years were invited to complete a written survey between 2011–3. Two cohorts of final year students were sampled due to the lower enrolments in the first graduating cohort of the medical school in 2011. Paper-based surveys and participant information sheets were distributed as questionnaires during mandatory sessions of the medical program, including small and large group sessions. Completed surveys were collected at the end of these sessions, after teaching activities had been conducted. For Year 4, data were collected following completion of the CR project. The survey questions were adapted from a questionnaire developed at a research-intensive medical school aimed at exploring student research experiences and attitudes to medical research [26]. Questions asked students about the type, duration, quality, and any outcomes from their research experience prior to and during medical school (closed-ended dichotomous and open-ended free text questions), intention to conduct research afterwards (quantitative estimate of likelihood), and level of agreement with attitudinal statements regarding research (5 scale Likert items- Strongly Agree, Agree, Neutral, Disagree, Strongly Disagree). The survey was slightly adapted for each year cohort to account for cumulative research experiences in each year of the program.

Data were analyzed using IBM SPSS Statistics version 19.0 (SPSS Inc., Armonk, New York). Data are expressed as mean (SD). Parametric assumptions were checked and appropriate non-parametric statistics used. Associations were determined using Spearman’s rho. Kruskal-Wallis tests with Bonferroni correction were used to: determine differences in responses between Years, between Pre-Clinical versus Clinical Years, and before completing (pre-CR) versus after completing (post-CR) Community Research projects. Significance was set at *p* < 0.05 for all analyses.

### Qualitative interview methods

At the time of survey data collection, students from all Years were invited to consent to a semi-structured interview. The interview centered on three open ended questions about the interviewee’s prior research experiences, and their perceptions of the barriers and of the facilitators to doing research during and after medical school. Purposive sampling of those who gave consent in the survey for further contact was used to sample views from all Years and levels of experience; from Year 1 students with no prior research experience to students who conducted doctoral research, to final year students who had completed the compulsory research program. This sampling strategy allowed for data across key variables, likely to affect the range of student experiences with research, to be gathered. Interviews were conducted by WH and SW between 2012–3, audiotaped and transcribed verbatim. Transcripts were independently reviewed by all authors for emergent themes, through a repeated process of reading for familiarization, identification and naming of *a priori* ideas as expressed by the interviewees, joint discussion to describe all identified themes, and then where appropriate, to resolve and combine conceptually similar themes and develop detailed interpretive descriptions of the new themes. Divergent views were sought, particularly from students with different experiences, compared with earlier themes, and either new unique themes developed or incorporated into existing themes and the descriptors revised. This process occurred concurrently with interviews so that sampling occurred until thematic saturation was reached. The authors range in research teaching experience from supervising individual students (all authors) to small group intensive teaching (SW, WH) to lecturing (SR) and curriculum coordination (WH), thus enhancing the data analysis and reducing the effect of particular teaching experiences. It is also important to note here that while authors had prior knowledge of SDT, the interview questions were not guided by SDT resulting in more authentically grounded results. Qualitative data analysis software (QSR Nvivo v10, Doncaster, Victoria, Australia) was used to systematically code all transcripts against the themes.

### Data integration and synthesis method

Free text responses in the questionnaire (n = 184 responses for all years) underwent independent thematic analysis by one of the authors (SW) and was compared with themes from the qualitative interviews through joint iterative discussion (WH, SR). No new insights or themes were identified from the free text responses.

Subsequently, the Self-Determination Theory domains of Autonomy, Competence and Relatedness were used as an analytic framework to interrogate and synthesize quantitative and qualitative findings. Content or thematic areas were identified from both datasets, compared and contrasted. Integration occurred at the point of final data interpretation, by identifying groupings of content areas, presenting both datasets in tables and generating narrative descriptions for each conceptual grouping [24]. These synthesized concepts were then used as the basis of a new explanatory model [27].

## Results

### Participant characteristics

The overall survey response rate was 71.8 % (579 of 806 potential respondents). Survey respondent age and gender by Year are shown in Table 1. Respondents did not differ significantly from non-respondents with regard to age (*χ*2(1) = 0.12; *p* = 0.73), with slightly fewer males (42.1 % vs 46.4 %; *χ*2(1) = 4.22; *p* = 0.04). Research experience by Year prior to medical school ranged from 5.0- 10.5 %, and of these, a majority (~60 %) had less than six months of experience. Prior experiences were mostly positive (84.3 % strongly agreed or agreed) with no differences by Year (*p* > 0.05).

**Table 1:** Participant demographics and prior research experience

	Year 1 (n = 181)	Year 2 (n = 119)	Year 3 (n = 86)	Year 4 (n = 56)	Year 5 (n = 137)	Total sample (n = 579)
Sex number (%)						
males	79 (43.6 %)	54 (45.4 %)	37 (43.0 %)	19 (33.9 %)	55 (40.1 %)	244 (42.1 %)
females	102 (56.4 %)	65 (54.6 %)	49 (57.0 %)	37 (66.1 %)	82 (59.9 %)	335 (57.9 %)
Age of student number (%)						
<20 yrs	144 (79.6 %)	67 (58.3 %)	81 (94.2 %)	49 (87.5 %)	115 (83.9 %)	456 (80.4 %)
21-24 yrs	18 (9.9 %)	39 (33.9 %)	1 (1.2 %)	4 (7.1 %)	16 (11.7 %)	78 (13.8 %)
25-29 yrs	6 (3.3 %)	7 (6.1 %)	2 (2.3)	--	--	15 (2.6 %)
30-34 yrs	4 (2.2 %)	--	--	2 (3.6 %)	1 (0.7 %)	7 (1.2 %)
35-39 yrs	4 (2.2 %)	--	2 (2.3 %)	--	1 (0.7 %)	7 (1.2 %)
40+ yrs	2 (1.1 %)	2 (1.7 %)	--	--	--	4 (0.7 %)
Prior research experience number (%)						
yes	15 (8.3 %)	6 (5.0 %)	9 (10.5 %)	3 (5.4 %)	10 (7.3 %)	43 (7.4 %)
no	165 (91.2 %)	109 (91.6 %)	77 (89.5 %)	53 (94.6 %)	127 (92.7 %)	531 (91.7 %)

Of the survey respondents, 156 consented to interview, representing more than 25 % of respondents in every Year. Of these, 23 were interviewed, including students from Pre-Clinical (n = 7) and Clinical Years (n = 16). Most were female (60 %), none had research experience prior to medical school, although 6 (26 %) had studied research as coursework. Six (26 %) had completed optional research, including doctoral, Honours and vacation scholarship research.

### Quantitative survey results

The stated likelihood of pursuing research after medical school, and level of agreement with particular attitudes towards research, were correlated with Year in the medical program (Pre-Clinical vs Clinical), and whether students had already completed the compulsory research component (pre-CR vs post-CR).

#### Intention to pursue research after medical school

Of the total sample, 42.3 % indicated greater than 50 % likelihood of pursuing research post-graduation (“*Future Intentions*”). A greater likelihood was associated with previous research experience (*r*_*s*_ = −0.27, *p* < 0.001), and with Year in the medical program (*r*_*s*_ = 0.28, *p* < 0.001), with more advanced students indicating higher *Future Intentions*. Pre-Clinical students indicated significantly less likelihood (*χ*^2^(1) = 42.89; *p* < 0.001) (48 %) compared to Clinical Years (64 %). Comparing pre-CR to post-CR students also yielded significantly weaker *Future Intentions* (*χ*^*2*^(1) = 37.36; 51 % vs. 66 %; *p* < 0.001, respectively). Post-CR students indicated a greater intention if their research experiences during medical school had been positive (*r*_*s*_ = −0.37, *p* < 0.001). Conversely, the more negative their research experience, the less likely Year 5 students would pursue post-graduation research (*r*_*s*_ = −0.43, *p* < 0.001).

#### Responses to attitudinal survey questions

Table 2 shows mean (SD) responses for all Years, compared between Pre-Clinical and Clinical Years, and between pre-CR and post-CR students, against each attitudinal statement. Associations between each attitude and *Future Intentions* were sought (for complete analyses see Additional file 1). In summary:

- *Conducting Research is advantageous for medical career* (*Career Advantage*), 89.1 % strongly agreed or agreed across all Years, with more agreement in the Clinical, than Pre-Clinical Years (*χ*^2^(1) = 20.07, *p* < 0.001), and in the post-CR compared to pre-CR students (*χ*^2^(1) = 19.38, *p* < 0.001).
- *Bureaucracy surrounding research is a significant deterrent* (*Bureaucracy*) indicated general neutrality (44.2 % strongly agreed or agreed). Clinical and post-CR students indicated greater agreement as compared to Pre-Clinical (*χ*^2^(1) = 26.14 and pre-CR (*χ*^2^(1) = 19.25, *p* < 0.001) students.
- *Research means a lower salary* (*Lower Salary*), there was neutrality (49.8 % indicated neutrality). Year 1 and 2 students were not different (*p* > 0.05) than Year 4 or 5 students and Clinical (*χ*^2^(1) = 3.20, *p* = 0.07) and CR (*χ*^2^(1) = 0.60, *p* = 0.44) cohorts were not different.
- *Research is important for keeping up to date in clinical field* (*Keeping Up to Date*), revealed general agreement (67.9 % strongly agreed or agreed), but greater agreement (*p* < 0.05) during year 4 as compared to year 5, with no differences (*p* > 0.05) between years 1, 2, 3 and 5 (*χ*^2^(4) = 8.92, *p* =0.06 or between Clinical (*χ*^2^(1) = 2.90, *p* = 0.09) or CR (*χ*^2^(1) = 0.45, *p* = 0.50) cohorts.
- *Research only suited to those wanting an academic career* (*Suited to Academic Career*), 63.7 % disagreed or strongly disagreed overall, with no differences (*χ*^2^(4) = 1.90, *p =* 0.75) between Years or Clinical (*χ*^2^(1) = 0.94, *p* = 0.33) or CR (*χ*^2^(1) = 0.93, *p* = 0.34) cohorts.
- *A research career is difficult to combine with a clinical career* (*Difficult with Clinical Career*), responses indicated neutrality (34.0 % strongly agreed or agreed) with no differences (*χ*^2^(4) = 4.04, *p* = 0.40) between Years or Clinical (*χ*^2^(1) = 2.69, *p* = 0.10) or CR (*χ*^2^(1) = 0.94, *p* = 0.33) cohorts.

**Table 2:** Responses to attitudinal questions for all Years, Pre-Clinical compared to Post-Clinical, Pre-CR compared to Post-CR stages in the medical program

	Yr 1	Yr 2	Yr 3	Yr 4	Yr 5	Pre-Clinical	Clinical	Pre-CR	Post-CR
Attitude question	Mean (SD)	Mean (SD)	Mean (SD)	Mean (SD)	Mean (SD)	Mean (SD)	Mean (SD)	Mean (SD)	Mean (SD)
Conducting Research is advantageous for medical career	1.8(0.7)^a^	1.9(0.6)^a^	1.7(0.7)^a,b^	1.5(0.6)^b^	1.6(0.7)^b^	1.8(0.7)	1.6(0.7)*	1.8(0.7)	1.6(0.7)*
Research only suited to those wanting an academic career	3.6(0.8)^a^	3.6(0.8)^a^	3.6(0.8)^a^	3.7(0.8)^a^	3.6(0.9)^a^	3.6(0.8)	3.6(0.8)	3.6(0.8)	3.6(0.9)
A research career is difficult to combine with a clinical career	2.9(0.9)^a^	2.9(0.8)^a^	3.1(1.0)^a^	3.1(1.0)^a^	3.0(0.9)^a^	2.9(0.9)	3.0(1.0)	3.0(0.9)	3.0(0.9)
Research is important for keeping up to date in clinical field	2.1(0.8)^a^	2.2(0.9)^a^	2.3(1.0)^a^	2.0(0.9)^a,b^	2.4(1.0)^a,c^	2.2(0.8)	2.3(1.0)	2.2(0.9)	2.3(1.0)
Research means a lower salary	3.2(0.8)^a^	3.1(0.8)^a^	2.8(0.8)^b^	3.1(0.8)^a,b^	3.1(0.8)^a,b^	3.1(0.8)	3.0(0.8)	3.1(0.8)	3.1(0.8)
Bureaucracy surrounding research is a significant deterrent	2.8(0.9)^a^	2.9(0.9)^a^	2.5(0.8)^b^	2.3(0.9)^c^	2.5(1.0)^c^	2.8(0.9)	2.5(0.9)*	2.8(0.9)	2.4(1.0)*
Likely to pursue research after degree (%)	50(26)^a^	45(26)^a,b^	59(29)^a,c^	64(27)^c^	67(27)^c^	48(26)	64(28)*	51(27)	66(27)*

Three statistical analyses were used to test group differences. Attitude questions were coded as 1 = Strongly agree, 2 = Agree, 3 = Neutral, 4 = Disagree, and 5 = Strongly disagree. *indicates statistical significance at *p* < 0.001 level for the 1) pre-clinical and clinical groupings as well as the 2) pre-CR and post-CR. 3) ^a,b,c^ indicate groupings for years that are not statistically different from one another at the *p* < 0.05 level

### Integrated qualitative interview and survey results

The final data integration into concepts, comprising a synthesis of the qualitative interview findings and quantitative survey results, and aligned to SDT domains of *Autonomy*, *Competence* and *Relatedness*, is summarized in Table 3. For clarity of presentation, a narrative summary of these eight integrated concepts is presented below with extracts of quotes. Illustrative quotes from students in different Years with different levels of clinical and research experience, are shown in Additional file 2.

**Table 3:** Integration of survey and interview findings, informed by Self-Determination Theory

SDT Domain	Integrated concept	Concept description	Survey	Illustrative interview response
			Significant associations with future intention to do research;* significant Year and stage differences*	
**AUTONOMY**	**Self & Time**	Managing self & time against uncertain outcomes. Similar to Bureaucracy, Financial, an extrinsic de-motivator. Students have not internalised a desire to do research. Research, and its unforeseen outcomes, is not a good fit with the immediate demands of study and lack of time for research.	Previous Research Experience	We had such a short period of time to work on a project, it’s hard to, hard to I guess pick a topic that’s interesting and at the same time feasible (105 Yr2)
Students initiate and regulate their research behaviors with a high degree of volition and a sense of choice.^19^				
			Total sample r_s_ = -0.27 All Stages	
			r_s_ = -0.16 Pre-clinical	
			r_s_ = -0.22 Clinical	
			r_s_ = -0.17 Pre-CR	I was just trying just to find my ground in terms of second year, had a big year, and then you come to the end of the holidays and you just want a break and then you find out that there’s a research opportunity there…. so it wasn’t the best timing (103 Yr 5)
			r_s_ = -0.22 Post-CR	
	**Career**	Career advantage is an extrinsic motivator but can be demotivating if not accompanied by a sense of a higher purpose, such as Clinical Relevance, the pursuit of scientific knowledge, or identification with research careers. Otherwise doing research feels inauthentic and forced.	Career Advantage	I find that in medicine, especially, there’s such heavy weight on people doing research, even though it doesn’t really reflect their clinical competence.….So I’m a little frustrated at that because I’d rather be doing research because I want to do it, rather than be doing it so my resume looks padded out (104 Yr 5)
			r_s_ = -0.38 (range = -0.24 – -0.49)	
			All Years, All Stages	
			Group differences: Mean(SD)	
			1.8(0.7) Pre-CR, 1.6(0.7)* Post-CR	
			1.8(0.7) Pre-Clinical, 1.6(0.7)* Clinical	
			Suited to Academic Career	
				The benefits are I think, that I’m hoping, that it will help me to get into specialty training easier. I was thinking of doing ophthalmology when I started and they only take in a couple of people every year, so I thought it would give me a good, good leg up into being a bit more competitive (101 Yr 2)
			r_s_ = 0.39 Yr4, r_s_ = 0.41 Yr5	
			r_s_ = 0.20 pre-CR, r_s_ = 0.36 post-CR	
			Difficult with Clinical Career	
			r_s_ = 0.30 Yr1, r_s_ = 0.16 Yr2, r_s_ = 0.24 Yr5	
			r_s_ = 0.22 Pre-Clinical,	
			r_s_ = 0.20 Clinical	
			r_s_ = 0.20 pre-CR, r_s_ = 0.23 post-CR	
	**Bureaucracy**	The bureaucracy and bother of doing research. Processes that hinder or assist the conduct of research, such as ethics. An extrinsic de-motivator which can be overcome if intrinsically motivated to do research, and if not seen to be due to lack of competence.	Bureaucracy	It ended up taking us nearly six months to get ethics approval, if not longer. And it was just a retrospective study and we were asking, you know, no names were going to be mentioned or anything like that…. It was really disheartening every time you’d send something in and you’d get rejected and it would be for some technicality that you didn’t even think existed (104 Yr5)
			r_s_ = 0.35 Yr4, r_s_ = 0.23 Yr5	
			r_s_ = 0.21 clinical, r_s_ = 0.25 post-CR	
			Group differences: Mean(SD)	
			2.8(0.9) Pre-Clinical, 2.5(0.9)* Clinical	
			2.8(0.9) Pre-CR, 2.4(1.0)* Post-CR	
			Research During	
			(r_s_ = -0.43 Yr5, where experience was positive during degree)	
				That’s it, like if there’s like a streamlined information [portal] of what research is going on and who needs help or what research you can take on and how to get in those positions. It kind of like feels, like I don’t know how to be involved even if I wanted to (111 Yr 4)
	**Financial**	Financial barriers and rewards. An extrinsic motivator, it can act as an immediate facilitator, but not deeply motivating in the longer term, especially for students who have internalised a desire to do research.	Lower Salary	I hate to say it, but it’s the finances. Research can be quite time intensive. Even with the summer res scholarship it doesn’t provide you with much income in return for the work that you’re doing (119 Yr 1)
			r_s_ = 0.21 Yr2 (range = -0.09 – 0.21)	
				I guess money’s a good thing, but I’m not trying to say money’s not my only thing that’s motivating me, but for other people, that kind of thing might be a push (122 Yr 1)
**COMPETENCE**	**Confidence**	At first, it seems research is only offered or suited to high performing students and is beyond one’s abilities. With experience there is increasing confidence in doing research, particularly when research support and supervision is provided, to reach a sense of achievement and mastery. A key example of increasing internalisation.	Previous Research Experience	I think coming straight out of school into the course and then in the first years you might get an email about some research opportunity. I guess because you have to apply and it’s a competitive process, I kind of thought, oh well, I won’t get it anyway or I haven’t got any experience yet, I’ll just have to wait until I get the experience. (108 Yr 5)
The need to be effective in interactions with research, the desire to exercise capacities, seek optimal challenges, and extend skills.^19^			Total sample r_s_ = -0.27, All Stages	
			r_s_ = -0.16 pre-clinical, r_s_ = -0.22 clinical	
			r_s_ = -0.17 pre-CR, r_s_ = -0.22 post-CR	
			(r_s_ = 0.27, All Years, All Stages)	
			Research During	
			(r_s_ = -0.43 Yr5, where experience was positive during degree)	
				I mean, it’s something I have accomplished myself so I can look at it and say, yeah I’ve accomplished this….I’ve shown, I’ve proven that I could do it. (101 Yr 2)
**RELATEDNESS**	**Clinical relevance**	Students enter medical school wanting to be clinicians; they are peripherally aware of research but cannot see its relevance until exposed to the realities of clinical practice. For those doing additional research, the sense of having added to scientific knowledge through their discoveries is highly motivating. Conversely, the notion of not being driven by practical relevance or discovery, but by venal aims such as career advancement, is demotivating.	Keeping Up to Date	But when we started our clinical years, we realised that sort of practice in the hospital isn’t sort of as cut and dried as the first few years of medicine. That sometimes clinical decisions are based on things that we don’t fully understand, so we have to base it on the best evidence out. So we started to realise, or I started to realise that research, what contributes to that and that’s helpful to further understand the decision we’re making, whether they’re the right ones (113 Yr3)
			(range = -0.26 – -0.32), All Years, All Stages)	
The need to establish close and secure attachments with others; feeling emotionally linked to and interpersonally involved in warm, caring relationships; connection with a ideals or goals held by a group; a sense of purpose.^19,21^				
				Another motivator is because people legitimately do want to be good doctors and they think that research is important for that. ….they feel they want to ask a clinical question and they truly do want to know the answer (107 Yr 5)
	**Research as a Social Activity**	Students are intrinsically motivated by social relationships; the image of research as lonely work, and poor quality relationships in research teams can be highly demotivating. However, good relationships and teamwork foster motivation and confidence.	Research During	When the school is selecting for medical students, they select I suppose friendly communicative kind of personalities, so maybe that’s why some of them are not so, they wouldn’t be so drawn to research which is maybe a more lonely kind of occupation (113 Yr 3)
			(r_s_ = - 0.43 Yr5, where experience was positive during degree)	
				The good part of it is everyone can contribute and different people have different ideas and different ways to look at things (117 Yr4)
	**Personal Relevance**	Increasing awareness of Clinical Relevance and of the researcher identity can be hastened by connection with a person to whom the student can relate, for example, a role model, mentor, or supervisor. These include researchers who teach or do clinical work, who counter the attitude that research is only suited to academically oriented students and that it cannot be combined with clinical work.	Only suited to academic career	I think I mostly heard about it from my grandfather because he was a virologist and he did work on influenza for 20 years, and so he would tell us stories about his research and it was just really, really fascinating to hear about someone discovering something that had never been seen by anyone else before.(101 Yr 2)
			r_s_ = 0.28 (range = 0.20– 0.41)	
			lowest agreement in Yr5(r_s_ = 0.41)	
			Difficult to combine with clinical career	
			r_s_ = 0.30 Yr1, r_s_ = 0.16 Yr2, r_s_ = 0.24 Yr5	
			r_s_ = 0.22 Pre-Clinical, r_s_ = 0.20 Clinical	
				Even before I’d decided I wanted to do medicine, I was interested in medical research, and I have a few family friends who are doctors and one of them said that a really good way of getting into medical research is also doing medicine and it was something I was considering, so that was actually one of the reasons I chose to do medicine (112 Yr 3)
			r_s_ = 0.20 pre-CR, r_s_ = 0.23 post-CR	
			Research During	
			(r_s_ = - 0.43 Yr5, where experience was positive during degree)	

*See Table 2 and Additional files 1 and 2, for detailed analyses. Statistically significant associations were sought between (1) research experience before, and during, the medical degree, and an intention to do research after the degree (negative associations reflect that research experiences are associated with higher future intention to do research), and (2) agreement with attitudes towards doing research, and an intention to do research after the degree (negative associations reflect that attitudes are associated with higher future intention to do research)

### Autonomy

#### Self & time

A low level of prior research experience, together with uncertainties about the nature of research, its potential outcomes, and distant future benefits, led to low motivation to do research, especially when considered against the immediate need to manage study demands on time, and limited curriculum time allocated to research.

I’ve got a bit of an interest area, given my background….but how that actually fits in doing, while undertaking my medical degree, I’m not entirely sure (114_Yr 3, Clinical, pre-CR)^1^

#### Career

Interviews were consistent with survey findings, with widespread acceptance that research activities provided a *Career Advantage*. But this was not due to being *Suited to Academic Career*, or *Difficult with Clinical Career*, but simply to improve chances of entering specialty training. *Career Advantage* alone, an extrinsic motivator, unaccompanied by a sense of its relevance to practice and the pursuit of knowledge, was *de*-motivating, as it felt forced and inauthentic.

The benefits are I think that I’m hoping that it will help me to get into specialty training easier…(101_Yr2, Preclinical, pre-CR)

I wish that wasn’t a reason why I want to do research but it sort of it is, yeah. (102_Yr5, Clinical, post-CR)

#### Bureaucracy

Research experience during medical school was associated with greater agreement with the deterrent effect of *Bureaucracy* and its processes, such as research ethics applications, on research motivation.

It was really disheartening every time you’d send something in and you’d get rejected and it would be for some technicality that you didn’t even think existed. (104_Yr5, Clinical, post-CR)

#### Financial

An extrinsic motivator, immediate financial barriers and inducements had more effect on motivation than longer-term effects on salary.

### Competence

#### Confidence

Lack of confidence in one’s ability to conduct research, and anxiety when faced with compulsory research was common. This was compounded by little prior research experience, and with optional activities only being offered to high performing students. But when research is successfully completed, confidence grows, together with increased *Future Intentions*.

I guess it really did open my eyes to the amount of work that goes into research and although it kind of puts you off…at the end of the day I’m quite proud of what we’d achieved (103_Yr5, Clinical, post-CR)

### Relatedness

#### Clinical relevance

While students acknowledged the connection of research with maintaining current knowledge, or *Keeping up to Date*, its real relevance was not felt until students were exposed to the uncertainties of clinical practice. Students aspire to be clinicians; the notion of a good doctor who is also a researcher emerges in the Clinical Years, and with mastery of research skills.

But when we started our clinical years, we realised that practice in the hospital isn’t as cut and dried as the first few years….so I started to realise that research, what contributes to that and to further understand the decision we’re making, whether they’re the right ones is important (113_Yr3,Clinical, pre-CR)

#### Research as a social activity

Positive experiences with group research counter early beliefs that research is lonely work. Students repeatedly cited the importance of working with colleagues, and a sense of satisfaction with joint achievements.

I guess it was comforting that it can, it’s doable and it’s not the most difficult thing, especially if you’re working as a group. (108_Yr5, Clinical, post-CR)

Conversely, differences between team members' expectations detracted from the research experience, although it led to learning about teamwork and one's place in a team.

#### Personal relevance

Role models, such as family members, teaching staff and supervisors could be positive motivators, potentially countering the notions of research being *Suited to Academic Career*, or *Difficult with Clinical Career.*

## Discussion

**Fig. 2:**
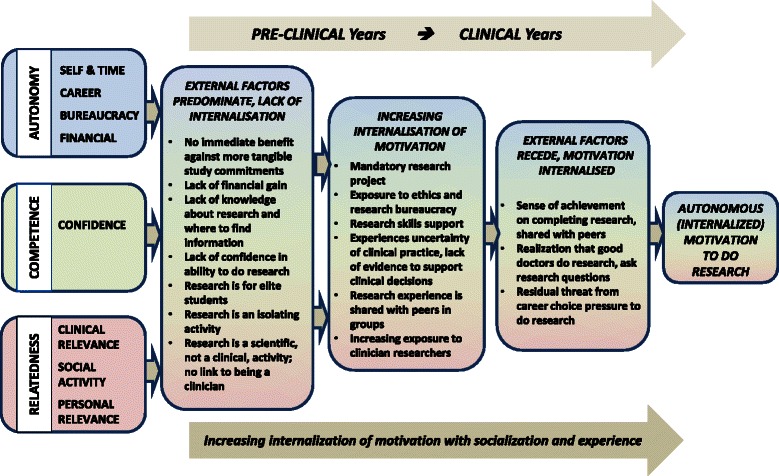
A proposed model for internalization of medical student motivations to do research

This study adds to previous work by adopting a theoretically informed and comprehensive mixed methods design to examine experiences of doing research in a “research naive” medical student population. Utilizing Self-Determination Theory, quantitative survey results were integrated with qualitative interview findings from all Years of a medical program to develop eight integrated concepts. From these, we propose a model for student motivations to do research at different stages during their medical degree (see Fig. 2). Our model suggests an increasing internalization of intentions to do research, with the balance of extrinsic (e.g. Self & Time, Finances, Bureaucracy) and intrinsic (e.g. Competence, Clinical and Personal Relevance) motivations shifting with cumulative clinical and research experience. Of practical relevance to curriculum designers, it suggests that motivation is increased in students who have commenced clinical clerkships, and who have completed a compulsory group research project. However, prior to doing research, students lack confidence, so predictably in our study, the quality of the research educational experience is associated with intention to continue doing research. This is consistent with studies where students who feel secure with critical appraisal and statistical skills have increased odds for involvement in research [28]. Our study goes further by including other factors such as financial concerns, time available and bureaucracy, which were considered barriers which were countered by the sense of achievement when research is completed. Career advancement was important, but in keeping with SDT, demotivating when in conflict with a more altruistic view of research, and with personal choice. A novel finding was the importance of research as a social activity for many students, and the motivating effect of group achievements where the experience was positive.

### Compulsory research activities in medical programs

Students who had completed the compulsory project reported significantly higher future research intentions. While this may appear counterintuitive to choice being a motivating influence, a key study finding, even in students who had conducted voluntary research activities, was that an anticipated lack of skills, and thus self-confidence in one’s ability, was highly de-motivating and countered any motivating effects of choice.

Medical school research experiences have been shown to predict later research achievement [9, 14, 29], but these studies have largely examined associations with voluntary activities by individual students. Few have demonstrated the effect of required research projects [15], and none of collaborative learning in research. Previous studies have also focused on graduate entry programs where prior research experience is more likely. In our study this was independently associated with greater intention to do research.

If students lack confidence and fail to see the relevance of research, they may not choose optional activities. Our findings support compulsory research, but given curricular time constraints [12] evidence based learning strategies should be used if research is to be part of the curriculum. Our participants cited: increased confidence following collaborative group work, provision of research skills support and supervision, and awareness of clinical relevance as being key reasons for increased motivation to do research. These are consistent with the SDT domains of Competence and Relatedness, and suggests practical strategies to optimize use of curricular time.

### Self-Determination Theory and curricular strategies

**Fig. 3:**
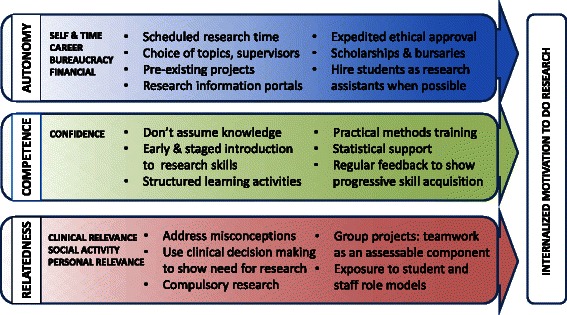
Curriculum strategies suggested by Self-Determination Theory and study findings to promote internalization of student motivations to do research

Figure 3 presents activities suggested by our model as likely to promote motivation to do research both during and after medical school. *Autonomy*, or choice, can be maintained in compulsory activities by offering students options for topics, supervisors and group composition, enhanced by elective research opportunities. As mentioned previously, in the SDT model of change, autonomy serves to facilitate an increased sense of competence, and competence alone is not sufficient to ensure change or adherence, but rather, must be associated with increased autonomy. Since external motivators are likely to be context dependent (e.g. money, time) these may not hold in the postgraduate context, making it all the more important to internalize motivation as undergraduates. Using arguments such as career advancement may enforce a perception of coercion, and together with constraints such as time and bureaucratic requirements, may de-motivate students. Feasible scheduling and assistance with navigating procedures, such as expedited ethics approval, may address concerns about juggling time and opaque bureaucratic processes.

Inadequate exposure and training in research methods have been shown as barriers to student involvement in research [30, 31]. A sense of *Competence* particularly in “research naive” students could be developed with staging and scaffolding expectations to do research, and emphasising achievements at each stage in the research process from formulating research questions to report writing. By gradually building confidence, the highly motivating effect of a sense of mastery when projects are completed could be maximized. It also suggests that to maximize uptake of optional research, opportunities should be offered *after* research training for all students in the program.

*Relatedness* occurs on several levels; at an interpersonal level, role models and supervisors can have a positive impact and correct misperceptions about clinical research. Effective teamwork which promotes collegiality and harnesses group member strengths to jointly achieve project goals can mitigate low confidence, and the impression of research as an isolating endeavor. Promoting well-functioning group work through guidance and assessment may be a key role for research mentors and supervisors.

In relation to a higher purpose, students enter medical school expecting to become clinicians. While medical students have positive attitudes to scientific methodology [11, 29, 32, 33], this does not relate to involvement in research until the link with clinical practice is made [29]. During clerkships, students begin to appreciate the uncertainty of clinical practice, and the importance of research to effective healthcare. Consistently translating research outcomes to realistic clinical practice throughout the program may thus enhance motivation. A belief that researchers cannot easily continue clinical practice has been found to be a barrier [34], although in this study students did not tend to see research as an advantage or a barrier to academic or clinical careers. This suggests the influence of senior role models in our program who successfully combine these roles. Invoking the higher and translational goals of research may also alleviate effects of extrinsic de-motivators such as financial and bureaucratic obstacles [35].

### Limitations

In our postulated model, we infer a change in motivation with cumulative clinical and research experiences, but a longitudinal cohort study is required to demonstrate this phenomenon. Other cross sectional surveys have reported increasingly positive attitudes towards research in Canadian undergraduate programs [30]. However, our theoretically informed and rigorous mixed methods approach, integrating a comprehensive sampling of qualitative and quantitative data, strengthens the empirical basis of the model.

The mixed methods design also addresses limitations in statistical analysis, which was exploratory in nature, to test associations and generate hypotheses for future work. Confounding variables were not tested, but follow-on regression analyses revealed that only attitudinal variables were associated with future intentions, adding nothing further to the analysis. A limited number of survey variables, and respondent homogeneity in age and prior research experience also did not support a stand-alone regression analysis.

Interviewees included students who had taken additional research activities such as voluntary vacation activities and higher research degrees, so their views may differ from other students by having an established desire to do research. However, all Years and the entire range of research experiences were purposively sampled, and thematic saturation achieved. Despite greater research engagement, these interviewees still strongly expressed a lack of confidence, but also the motivating effect of having completed research successfully. As they are a small cohort in a new medical school, their interview findings are not presented separately, but a comparison could be a focus for further study.

Generalizability of findings is limited but the detailed description of study context and use of established theory suggests that findings could be transferrable and tested in other medical programs, particularly research naive student populations.

## Conclusions

Self-Determination Theory is useful for better understanding the motivations of individuals faced with complex choices. A mixed method study design, together with a theoretically informed analysis, has provided evidence for the motivating effects of Competence and Relatedness in relation to medical students doing research. This suggests that curriculum strategies such as well-supported compulsory research activities, conducted in groups, may lead to more effective learning about research, and promote future career involvement in research.

## Additional files

Additional file 1:
**Associations between response to attitudinal questions and intention to do research against age, Pre-Clinical, Post-Clinical, Pre-CR and Post-CR stages in the medical program.**
Additional file 2:
**Illustrative quotes from different student Years for each integrated concept, aligned to Self Determination Theory.**

## Footnotes

1 The attribution for each quote comprises a unique identifier for each participant, and the participants’ stage in the 5-year medical program. For example, 114_Yr3 is Participant 114, in Year 3.
