# Supplementary material for: Motivating medical students to do research: a mixed methods study using Self-Determination Theory
Source: BMC Med Educ. 2015 Jun 2;15:95. doi: 10.1186/s12909-015-0379-1 (PMC4486085; doi:10.1186/s12909-015-0379-1)
Supplement: Additional file 1: — Associations between response to attitudinal questions and intention to do research against age, Pre-Clinical, Post-Clinical, Pre-CR and Post-CR stages in the medical program. [file 12909_2015_379_MOESM1_ESM.docx]

Additional file 1. **Associations between response to attitudinal questions and intention to do research against age, Pre-Clinical, Post-Clinical, Pre-CR and Post-CR stages in the medical program.**

| Group | Research prior to medical school | Age | Conducting Research is advantageous for medical career | Research only suited to those wanting an academic career | A research career is difficult to combine with a clinical career | Research is important for keeping up to date in clinical field | Research means a lower salary | Bureaucracy surrounding research is a significant deterrent |
| --- | --- | --- | --- | --- | --- | --- | --- | --- |
| Yr 1 | -0.132,  *p* = 0.14 | 0.211,  *p* = 0.02* | -0.235,  *p* < 0.01** | 0.208,  *p* = 0.02* | 0.304,  *p* < 0.001*** | -0.321,  *p* < 0.001*** | 0.078,  *p* = 0.38 | 0.030,  *p* = 0.74 |
| Yr 2 | -0.170,  *p* = 0.02* | 0.003,  *p* = 0.97 | -0.493,  *p* < 0.001*** | 0.204,  *p* < 0.01** | 0.156,  *p* = 0.03* | -0.305,  *p* < 0.001*** | 0.214,  *p* < 0.01** | 0.114,  *p* = 0.11 |
| Yr 3 | -0.145,  *p* = 0.21 | 0.114,  *p* = 0.33 | -0.342,  *p* < 0.01** | 0.271,  *p* = 0.02* | 0.111,  *p* = 0.35 | -0.299,  *p* = 0.01* | 0.040,  *p* = 0.74 | 0.199,  *p* = 0.09 |
| Yr 4 | -0.246,  *p* = 0.09 | -0.181,  *p* = 0.22 | -0.442,  *p* < 0.01** | 0.390,  *p* < 0.01** | 0.212,  *p* = 0.15 | -0.323,  *p* = 0.03* | -0.134,  *p* = 0.37 | 0.351,  *p* = 0.01* |
| Yr 5 | -0.200,  *p* = 0.02* | 0.091,  *p* = 0.31 | -0.385,  *p* < 0.001*** | 0.410,  *p* < 0.001*** | 0.235,  *p* < 0.01** | -0.294,  *p* = 0.001** | -0.019,  *p* = 0.83 | 0.233,  *p* < 0.01** |
| Pre-Clinical | -0.161,  *p* = 0.02* | 0.125,  *p* = 0.06 | -0.363,  *p* < 0.001*** | 0.203,  *p* < 0.01** | 0.217,  *p* = 0.001*** | -0.313,  *p* < 0.001*** | 0.137,  *p* = 0.04* | 0.070,  *p* = 0.23 |
| Clinical | -0.221,  *p* = 0.001*** | 0.071,  *p* = 0.52 | -0.347,  *p* < 0.001*** | 0.359,  *p* < 0.001*** | 0.203,  *p* < 0.01** | -0.277,  *p* < 0.001*** | -0.021,  *p* = 0.75 | 0.210,  *p* = 0.001*** |
| Pre-CR | -0.165,  *p* < 0.01** | 0.073,  *p* = 0.14 | -0.363,  *p* < 0.001*** | 0.225,  *p* < 0.001*** | 0.201,  *p* < 0.001*** | -0.289,  *p* < 0.001*** | 0.066,  *p* = 0.21 | 0.070,  *p* = 0.24 |
| Post-CR | -0.217,  *p* < 0.01** | 0.049,  *p* = 0.52 | -0.346,  *p* < 0.001*** | 0.385,  *p* < 0.001*** | 0.229,  *p* < 0.01** | -0.264,  *p* <0.001*** | -0.091,  *p* = 0.23 | 0.248,  *p* = 0.001*** |
| Total  Sample | -0.260,  *p* < 0.001*** | 0.019,  *p* = 0.68 | -0.383,  *p* < 0.001*** | 0.278,  *p* < 0.001*** | 0.226,  *p* < 0.001*** | -0.265,  *p* < 0.001*** | 0.030,  *p* = 0.51 | 0.081,  *p* = 0.08 |

* indicates significance at the *p* < 0.05 level. **indicates significance at the *p* < 0.01 level. *** indicates significance at the *p* < 0.001 level. Associations are r_s_ from Spearman’s rho. Positive associations indicate that LESS agreement with the attitude question was associated with higher percent intention to pursue research following degree. Negative associations indicate MORE agreement with the attitude question was associated with higher percent intention to pursue research following degree.
