# Supplementary material for: Motivating medical students to do research: a mixed methods study using Self-Determination Theory
Source: BMC Med Educ. 2015 Jun 2;15:95. doi: 10.1186/s12909-015-0379-1 (PMC4486085; doi:10.1186/s12909-015-0379-1)
Supplement: Additional file 2: — Illustrative quotes from different student Years for each integrated concept, aligned to Self Determination Theory. [file 12909_2015_379_MOESM2_ESM.docx]

## Additional file 2. Illustrative quotes from different student Years for each integrated concept, aligned to Self Determination Theory

| **SDT** | **Concept** | **Illustrative interview response** |
| --- | --- | --- |
| **AUTONOMY** | **SELF & TIME** | So I don’t want to waste any more time spending time doing something and then not finishing it (101_Yr2, Preclinical, pre-CR)  I’ve got a bit of an interest area, given my background….but how that actually fits in doing, while undertaking my medical degree, I’m not entirely sure (114_Yr 3, Clinical, pre-CR)^[[1]](#footnote-1)^  [I was] just trying just to find my ground in terms of second year, had a big year, and then you come to the end of the holidays and you just want a break and then you find out that there’s a research opportunity there…. so it wasn’t the best timing (103_Yr5, Clinical, post-CR)  I heard about that and for me, I don’t think that I’m going to spend my whole life doing research so I didn’t thinking taking a year would, was going to help me at this point right now. (104_Yr5, Clinical, post-CR) |
|  | **CAREER** | I think it’s good for your resume…people think that you went into that topic a lot more, understood it a lot more, and then, hence you will be more acceptable in that career. (122_Yr1, Preclinical, pre-CR)  The benefits are I think that I’m hoping that it will help me to get into specialty training easier, and I was thinking of doing ophthalmology when I started and they only take in a couple of people every year, so I thought it would give me a good, good leg up into being a bit more competitive (101_Yr2, Preclinical, pre-CR)  So they need to find this tiny little niche where they can get, just use it to get some sort of publication….. It’s not something that they’re particularly interested in or passionate about or something that will really help the world, it’s just something that they can put on their CV….I wish that wasn’t a reason why I want to do research but it sort of it is, yeah. (102_Yr5, Clinical, post-CR)  I think it’s really sad in a way that they’ve [a specialty program] made it mandatory to do research because I think it encourages a lot of rubbish research to be done. People who aren’t motivated or don’t have an interest in clinical question they actually want to answer (106_Yr5, Clinical, post-CR) |
|  | **BUREAUCRACY** | It ended up taking us nearly six months to get ethics approval, if not, longer. And it was just a retrospective study and we were asking, you know, no names were going to be mentioned or anything like that….but it still took so long and so much effort on all our parts. It was really disheartening every time you’d send something in and you’d get rejected and it would be for some technicality that you didn’t even think existed. (104_Yr5, Clinical, post-CR)  The mechanics of starting a research project can take a lot of time, unless somebody hands you a premade one. If you’re setting one up yourself, it’s really quite labour intensive (101_Yr2 Preclinical, pre-CR)  I really don’t know what it entails. I don’t really see what I have to do, just not a lot of information. I’m really not sure what it entails (118_Yr1 Preclinical, pre-CR)  And if [there’s] an easy way to get involved, or know how to get involved, I guess. That’s it, like if there’s like a streamlined information [portal] of what research is going on and who needs help or what research you can take on and how to get in those positions. It kind of like feels, like I don’t know how to be involved even if I wanted to (111_Yr4, Clinical, post-CR) |
|  | **FINANCIAL** | I guess money’s a good thing, but I’m not trying to say money’s my only thing that’s motivating me, but for other people, that kind of thing might be a push. (122_Yr1 Preclinical, pre-CR)    I hate to say it, but it’s the finances. Research can be quite time intensive. Even with the summer research scholarship it doesn’t provide you with much income in return for the work that you’re doing (119_Yr1 Preclinical, pre-CR)  Well, I can’t do it without a scholarship because I can’t survive. So, but that ended up coming through, so because I had that scholarship then I could, I worked on the weekends and, and I had [government student] benefits as well, so that was okay (101_Yr2 Preclinical, pre-CR) |
| **COMPETENCE** | **CONFIDENCE** | I think coming straight out of school into the course and then in the first years when you might get an email about some research opportunity… I guess because you have to apply and it’s a competitive process, I kind of thought, oh well, I won’t get it anyway, I haven’t got any experience yet, I’ll just have to wait until I get the experience. It kind of feels like if you go into it without any research experience beforehand, and you’re doing it not as part of your course, then there’s a little bit of an expectation that you may not be able to live up to. That was how I felt in the first few years. (108_Yr5, Clinical, post-CR)  But…you have to have a Distinction average, so you’re not going to get it, so it’s kind of like, most students don’t go for it, on that basis, I think. (111_Yr4, Clinical, post-CR)  I wouldn’t say I feel confident about it. It’s quite sporadic, like in between all the other things which are given heaps more time. (113_Yr 3,Clinical, pre-CR)  I suppose a staged kind of introduction to basic sort of research activities like data entry and that would building onto other things and rebuild that relationship with people in a way that people might, so they would gradually increase their confidence (115_Yr4, Clinical, post-CR)  I think had I not had any research experience I would feel, I wouldn’t feel inclined to put my hand up to do any further research as an intern or registrar. I guess because if you had no idea what’s going on, you’d feel a little I guess, embarrassed not to know anything like how to start a research project or what’s actually involved or expected. So I think having done a little bit of research and a couple of projects, I’d be more inclined to seek out opportunities whilst working. (110_Yr5, Clinical, post-CR) |
|  |  | I mean, it’s something I have accomplished myself, so I can look at it and say, yeah I’ve accomplished this, I’ve stuck at it, you know, and that I was able to do it. I’ve shown, I’ve proven that I could do it (101_Yr2, Preclinical, pre-CR)  I guess it really did open my eyes to the amount of work that goes into research and although it kind of puts you off, you stand back at the end of the day and I’m quite proud of what we’d achieved (103_Yr5, Clinical, post-CR) |
| **RELATEDNES** | **CLINICAL RELEVANCE** | That’s a possibility, but right now I’m just excited to be working with patients…. that’s one of my favourite parts of the week, so right now it has to be something really appealing about research, that would take me away from the clinical environment (122_Yr1, Preclinical, pre-CR)  But when we started our clinical years, we realised that practice in the hospital isn’t as cut and dried as the first few years of medicine. That sometimes clinical decisions are based on things that we don’t fully understand, so we have to base it on the best evidence out. So we started to realise, or I started to realise that research, what contributes to that and to further understand the decision we’re making, whether they’re the right ones is important (113_Yr3,Clinical, pre-CR)  It’s important for everyone to get a taster. It’s not everyone’s cup of tea, but in the long run, it’s relevant to everyone’s practice, and we all have to understand that (119_Yr 1, Preclinical, pre-CR)  Like as a student, when you get a compulsory research thing, then you kind of go, you moan and groan, but like I realised the importance of it after I’ve done it and after I’ve moaned and groaned (111_Yr4, Clinical, post-CR) |
|  | **RESEARCH as a SOCIAL ACTIVITY** | One thing occurred to me was that when the school is selecting for medical students, they select I suppose friendly communicative kind of personalities, so maybe that’s why some of them are not so, they wouldn’t be so drawn to research which is maybe a more lonely kind of occupation (113_Yr3)  It depends what you mean by research….I mean I don’t want to be the type of researcher who spends their whole time in the lab working out a particular genome (120_Yr1, Preclinical, pre-CR)  Obviously you won’t be in charge of the research team, but you’d had experience working with the research team…it would give you exposure to teamwork and working together and that’s good too (122_Yr1,Preclinical, pre-CR)  I guess it was comforting that it can, it’s doable and it’s not the most difficult thing, especially if you’re working as a group. (108_Yr5, Clinical, post-CR) |
|  | **PERSONAL RELEVANCE** | It’s pretty early days, I’d still say that my interest in doing research……traditional research outside the program is low….and maybe if there was something….I suppose if there was the opportunity, I’d look at it, but I can’t imagine jumping at the chance at moment (120_Yr1, Preclinical, pre-CR)  I think I mostly heard about it from my grandfather because he was a virologist and he did work on influenza for 20 years, research on influenza and so he would tell us stories about his research and it was just really, really fascinating to hear about someone discovering something that had never been seen by anyone else before (101_Yr2, Preclinical, pre-CR)  Like it’s always I guess open for us to go see I don’t know, like a lecturer or one of the consultants for our teams or something like that but I think as a student, you feel a bit hesitant to approach, someone so senior about research, just because we haven’t really had much experience. So it’s kind of hard to, yeah, broach that idea.(105_Yr5, Clinical, post-CR)  I also like research, like going through all the literature and viewing it and writing and putting things together and putting together the research to come to a conclusion and end. I’ve always enjoyed that, so the constant reinforcement of it was more that you can do this and it’s a good idea so I never felt pressured, because it was something I always intended to do. (107_Yr3,Clinical, pre-CR)  And because I read up on it again for my purpose, for my own needs, I paid more attention (115_Yr4, Clinical, post-CR) |

The attribution for each quote comprises a unique identifier for each participant, and the participants’ stage in the 5 year medical program. For example, 114_Yr3 is Participant 114, in Year 3.

1. [↑](#footnote-ref-1)
